# Electronic prescription system requirements: a scoping review

**DOI:** 10.1186/s12911-022-01948-w

**Published:** 2022-09-03

**Authors:** Marjan Vejdani, Mehdi Varmaghani, Marziyhe Meraji, Jamshid Jamali, Elaheh Hooshmand, Ali Vafaee-Najar

**Affiliations:** 1 Student Research Committee, Mashhad University of Medical Sciences, Mashhad, Iran; 2 Department of Health Economic and Management Sciences, School of Health, Mashhad University of Medical Sciences, Daneshgah 18, Mashhad, Khorasan Razavi Iran; 3 Social Determinants of Health Research Center, Mashhad University of Medical Sciences, Mashhad, Iran; 4 Department of Health Information Technology, School of Paramedical Sciences, Mashhad University of Medical Sciences, Mashhad, Iran; 5 Department of Biostatistics, School of Health, Social Determinants of Health Research Center, Mashhad University of Medical Sciences, Mashhad, Iran

**Keywords:** Electronic prescription, Requirements, Standards

## Abstract

**Background:**

An electronic prescription system is a mechanism that has long been implemented in many countries around the world. In the present study, we reviewed the requirements, standards, and features of an electronic prescription system for its correct and accurate execution.

**Methods:**

This scoping review was conducted according to the PRISMA-SCR (Preferred Reporting Items for Systematic Reviews and Meta-Analyses Extension for Scoping Reviews). A comprehensive literature search was performed with the related keywords in Web of Science, PubMed, Scopus, and ProQuest with no time limit. The selection of papers was based on inclusion criteria. After removing duplicates, reviewing titles, abstracts, and full-text, 13 articles were included in the analysis.

**Results:**

Electronic prescription system requirements extracted from the studies: Patient data, Patient selection or identification and data access, Drug Selection, Security, Privacy and administration, Transparency and accountability, Interoperability and communication, Monitoring, report, reminder, and renewals, Feedback at the prescriber level, Infrastructure: Computer equipment, Awareness of physicians and System support, Patient education and information, Usability, Standards, History of Medications / Current Medications, Data transfer and storage, Alerts and other messages to prescribers, and filtering of user-selectable alerts for possible prescription problems and Decision support.

**Conclusions:**

The results of this study showed that the electronic prescription systems have several functional and technical capabilities that can provide significant benefits to all system’s stakeholders, including service providers, drug distributors, patients, and insurance organizations if used correctly.

## Background

Electronic Prescribing is a broad term used to define either computer-based systems to write drug prescriptions, or comprehensive systems supporting the prescribing process [1]. The following are some of the benefits of e-prescription: improving the quality of health care services, increasing the efficiency and effectiveness of prescribing and dispensing medications, reducing medication errors, lowering health care costs, increasing patient safety, improving prescription, saving time for doctors, pharmacists, and patients, preventing adverse drug reactions, more precise dosage, monitoring how prescription drugs are prescribed, prescription abuse, and overprescribing [2–5]. The main steps to create and manage the electronic prescriptions are (1) a user (admin or doctor) sign-on, (2) the physician identifies the patient in the electronic prescription system. At this stage, the physician reviews the available data, (3) warnings and recommendations should be considered in the three activities of the electronic prescribing process, such as drug selection, parameter entry, and prescription signing, (4) the approved prescription was sent directly or indirectly to the pharmacy for distribution [6]. Systems must be able to interact with each other to share important information between health care centers. Multiple systems' inability to communicate information in standard forms and vocabularies has become a barrier to efficient electronic transcription deployment, highlighting the need for standards in the area of electronic transcription [7]. E-prescribing standards, like any structural component of health care, should be based on the extent to which they enable improvements in health care processes and outcomes [8]. All systems that are capable of electronically transmitting prescriptions share certain characteristics such as a need for connectivity. Most systems will require dedicated telephone lines or broadband Internet connectivity, a potential problem in more remote areas. All of them will require a computer, modem to connect to the telephone (usually dedicated digital subscription line) or Internet (via digital subscription line or cable), and likely a router. The need for connection may establish a single point of failure, rendering the whole system useless in the event of a malfunction, especially for applications that operate as application service provider systems [9]. Studies showed that pharmacopeia and insurance information standards to achieve the desired results in electronic prescribing are necessary but not sufficient, so more work needs to be done on medication or pharmacopeia and insurance information standards to increase patient safety. Additionally, incentives should be given to enable good communication between organizations engaged in prescription and payment for drugs, so that the full advantages of the electronic prescribing system may be realized via timely, patient-centered communication across these systems [8]. Wang's research demonstrates that currently existing electronic prescription systems lack a number of functional characteristics that might have a substantial impact on patients' health and expenditures. More importantly, these deficiencies varied a great deal among the systems studied. They suggest that standards for electronic prescribing should include a set of minimal functional capabilities; because would guarantee a minimal level of support for patient safety and protect against biases of third-party [10].

Regarding we did not find an analysis of the requirements for the optimal operation of electronic prescribing systems so this study was conducted to identify the requirements of the electronic prescribing system.

## Methods

This scoping review was conducted by the PRISMA-SCR (Preferred Reporting Items for Systematic Reviews and Meta-Analyses Extension for Scoping Reviews) [11] and Arksey and O’Malley’s framework [12]. The framework includes 1. identifying the research questions, 2. identifying relevant studies, 3. study selection, 4. data charting, 5. collating, summarizing, and reporting the results. According to this framework, comprehensive coverage of a subject should be provided, and its purpose is to identify all relevant literature without considering the design of the study. This study includes the following steps:

- Step 1: Identifying the research questions

Consultation and exchange with the research team were used to identify the main research question. The research questions were designed in such a way that include requirements, standards, and key features of the electronic prescribing system. In other words, the questions were selected in accordance with the objectives of the research.

Research questions include:

- What are the features of the electronic prescribing system?
- What are the requirements and standards of an electronic prescribing system?
- What are the recommendations for the electronic prescribing system?

The inclusion criteria for studies were:

(1) Quantitative, qualitative, mixed-method, and review published studies, original and gray texts including thesis, proceedings, and reports, (2) studies published in English, and (3) studies whose full text was available for data extraction.

The exclusion criteria were as follows: (1) studies in languages other than English, (2) studies whose full text was not available.

- Step 2: identifying the relevant studies

The main researcher and an expert person with a background in review studies (an experienced health economist) helped develop a keyword search protocol. The two researchers independently conducted extensive and comprehensive searches in the electronic databases of Web of Science, PubMed, Scopus, and Proquest, regardless of the period up to date 2021-06-19 to identify relevant studies. The following keyword combination was used for the search. Moreover, search terms were customized for each database individually.

"Prescribing Electronic", "Electronic Transmission of Prescriptions," "Electronic Prescription", "Electronic medical prescription", "Electronic prescription system", "Electronic Prescription Service", "On-line prescribing", "Characteristics", "Requirements", "Concept", "Features", "Standards", "Recommendation", "guidelines", and "criteria"

We entered the results into referral management software (EndNote X8.2) and removed duplicates. Two team members reviewed and verified the search results. All search procedures and results were documented.

- Step 3: Study selection

**Fig. 1:**
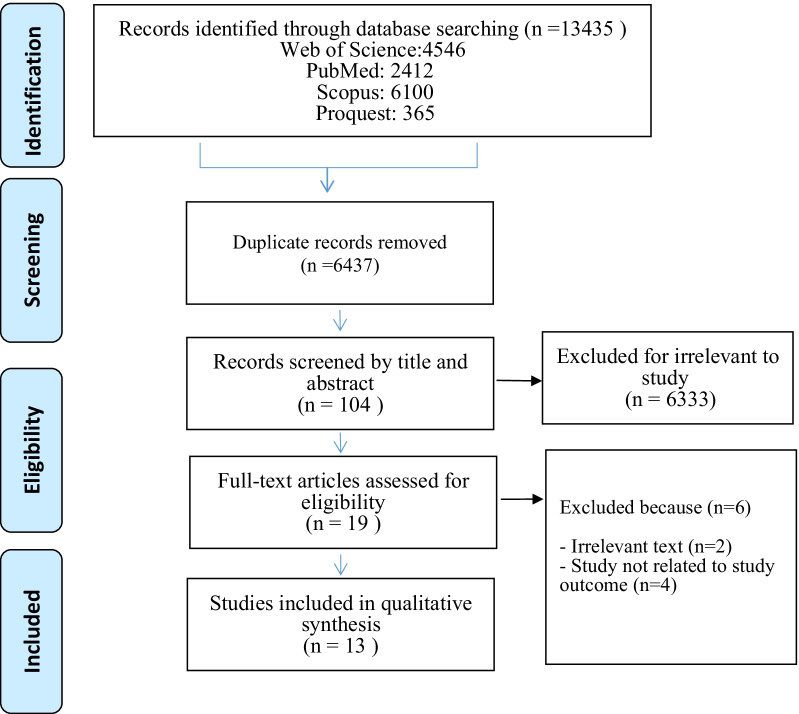
Flowchart of study retrieval and selection process (adapted from PRISMA)

After implementing the search strategy, the first stage of the selection process was performed; two researchers independently reviewed the titles and abstracts of all studies and screened them based on inclusion and exclusion criteria. A third party resolved the disagreement regarding the Competency of the documents. To assess how the screening process is progressing, a regular discussion among research team members was conducted. Unrelated studies were discarded and the full text of the remaining studies was reviewed. Two individuals independently reviewed the full text of these studies to confirm their relevance (Fig. 1).

- Step 4: data charting

The data extracted from each study include the following: title, author (s), date of publication (year), place of study, type of study, type of document, and key findings.

- Step 5: collating, summarizing, and reporting the results

This step includes gathering, summarizing, and reporting the results. To create and develop a framework for summarizing and combining data and summarizing results, researchers should prioritize specific aspects of the literature [12]. This study used a thematic analysis approach to collating and summarizing the findings. First, one researcher (Ma.V.) read all the records, annotated them, and identified topic categories. the same researcher re-read and finaled all of the records listed under each topic category. To establish trustworthiness, a second researcher (E.H.) verified the analysis for the records listed.

## Results

The data collected from the databases were as follows: 4546 records from Web of Science, 2412 records from PubMed, 6100 records from Scopus, and 365 records from Proquest. A total of 13,435 original articles and gray texts were found. 6998 records were duplicates and had been removed. By examining the titles of the texts, it was determined that 6333 entries did not meet the inclusion requirements and were thus eliminated. The remaining 104 items were evaluated for their titles and abstracts. 19 full-text papers were retrieved and evaluated, and six records were excluded due to conflicts with the research's goal. Also the full texts of 4 of them were not found. Finally, 13 papers were chosen for the analysis of the complete review. The oldest study was published in 2000, and the most recent study was published in 2015. Among the studies reviewed, 8 studies were original articles [10, 13–19], 3 studies were reported [20–22], 1 study was editorial [23] and 1 study was a review article [24]. 6 studies (%78) were conducted in the US [10, 13, 14, 20–22] and the rest of the studies were carried out in various other countries, as listed in Table 1.

**Table 1:** General characteristics of the studies included in this research

Study	Research location	Document	Type of study	Participants
Edward P. Elizabeth A, 2000 [23]	Georgia	Editorial	–	–
Douglas S. Bell, Richard S. Marken, et al., 2004 [14]	USA	Original article	Qualitative	Medicine, nursing, pharmacy, managed care, pharmacy benefit management, consumer advocacy, medical informatics, health care oversight, health care quality and safety, and health economics
Douglas S. Bell, Shan Cretin, et al. 2004 [13]	USA	Original article	Qualitative	E-prescribing vendors
Jonathan M. Teich, Jerome A. Osheroff, et al., 2005 [20]	USA	Report	–	–
C. Jason Wang, Richards S. Marken, et al., 2005 [10]	USA	Original article	Descriptive	Candidate vendors of electronic prescribing systems,
Robyn Tamblyn, Allen Huang, et al., 2006 [15]	Canada	Original article	Cohort	Primary care physicians and consenting patients
Robert S. Gerstle, Christoph U. Lehmann, et al., 2007 [21]	USA	Report	–	The pediatrician in the ambulatory setting
Douglas S. Bell, Anthony J. Schueth, et al., 2007 [22]	USA	Report	–	Physicians
Michelle Sweidan, Margaret Williamson, et al., 2010 [16]	Australia	Original article	Qualitative	Multidisciplinary expert group
Mohamed El, Houseny El, et al., 2011 [17]	Egypt	Original article	Cross-sectional	Physicians from different specialties, pharmacy staff (pharmacists and assistant pharmacists), nurses, and outpatients
Mahnaz Samadbeik, Maryam Ahmadi, et al., 2013 [24]	Iran	Review a	Review	–
Stephen Ward, Max Watson, 2013 [18]	Northern Ireland	Original article	Mixed-methods	Different professionals involved (consultant, registrar, ward manager, staff nurse, and pharmacist)
Ömer Gider, Saffet Ocak, et al., 2015 [19]	Turkey	Original article	Cross-sectional	Physicians

After conducting the research steps, electronic prescription system Features, requirements, standard, recommendations or capabilities were extracted from the studies: Patient data, Patient selection or identification and data access [10, 13, 14, 16], Drug Selection [13–16, 18, 24], Security, Privacy and administration [10, 14–16, 18], Transparency and accountability [10, 14, 16], Interoperability and communication [18], Monitoring, report, reminder, and renewals, Feedback at the prescriber level [10, 13, 14, 16], Infrastructure: Computer equipment, Awareness of physicians and System support, Patient education and information, Usability [10, 13, 14, 16–18, 20, 21, 23], Standards [20, 22, 24], History of Medications / Current Medications [10, 14], Data transfer and storage [10, 14], Alerts and other messages to prescribers, and filtering of user-selectable alerts for possible prescription problems and Decision support [13–16, 18] (Table 2).

**Table 2:** Features, requirements, standards, recommendations, and capabilities for the electronic prescribing system

Number	Features, requirements, standards, recommendations, or capabilities	Item	
1	Recommendations, requirements, and capabilities	Patient data, Patient selection or identification and data access [10, 13, 14, 16]	Import and export of demographic data and patient identification by the system, Import and export of patient clinical data from / to external sources, manual entry of the patient identification and demographic data when importing information from a practice management system is not possible, merge duplicate records created for the same patient, search for an individual patient by partial name and demographic data contained in the patient record, use the national Individual Healthcare Identifier when it becomes available, records and displays the names and reason for use (if known) of the complementary medicines used by the patient, records and displays a list of illicit/street drugs used by the patient, possibility to change or discontinue a current medication, recording the date, the prescriber name and the reason/s for the change or discontinuation, records and displays clinical information including past medical history, physical examination, undifferentiated problems, provisional or confirmed diagnoses and laboratory and other investigations, and the date of those measurements [16, 24], link and de-link records for patients within the same family, records and displays a patient's pregnancy status or if the patient is currently breastfeeding, records and displays a patient's status as an elite athlete, records, displays and allows updating of the patient's allergies and drug intolerances, records and displays preventative and non-pharmacological management measures in a format for decision support, create a clinical management plan for a patient, in a standard and configurable format that can be automatically populated with data in the patient record [16] Capability to import patient identity and demographic information from electronic medical records (EMRs) or practice management systems (PMSs) [16], Integration with electronic health record items such as a list of issues and test findings [16, 25, 26], Possibility of access to the relevant electronic prescription if the unique version number in the printed prescription is entered by the pharmacist [15], Data transfer to inpatient, retail, and/or postal pharmacies [13]
2	Features, recommendations, requirements, and capabilities	Drug Selection [13–16, 18, 24]	Providing information on drug formulations [10, 16, 18, 20, 23, 24], creating a complete list of active drugs [24], registration of drug indications and activation of decision support, quickly and accurately select the most appropriate problem/diagnosis [16], patient support during prescription and active promotion in using the appropriate drugs [24], displays recommended therapeutic options for the selected problem/diagnosis, and differentiates those options that are contraindicated for the patient, define 'order sets' that can be used to easily and quickly manage common problems, immediate access to the meaning of any symbols, icons or special fonts throughout the prescribing process, access to the Therapeutic Goods Administration approved product information† for medicines, access to independent evidence-based information about medication effectiveness and safety, if medications are listed on the Pharmaceutical Benefit Scheme then displays related details including costs [16], providing information on the availability of cheaper drugs and medically appropriate treatment alternatives [17, 24], proposing automatic supply of alternative drugs in case of prescribing non-formulated drugs [23], providing a list of available strengths and forms for the selected medication, providing a list of predefined dosage regimens that may be suitable for the selected medication, providing information on adult doses and children's doses [16], providing age alerts [24], access to dose calculators to assist with dosage calculations e.g. based on weight or age, presentation options for medication selection order to difficult to erroneously select a drug with a similar name, select a medication by drug class, when a brand name is selected, system displays the generic name (active ingredient/s) of the medication, enter medicines that are not on the medication list in the system, in a free text format, specify the length of a course of medicine on the prescription by prescriber, notes on taking or not taking a medicine on day/s of the week, easily re-order a medication from items already on the medication list, creating a prescription that conforms to Pharmaceutical Benefits Scheme† requirements [16], System reminders when drugs are ignored, Ability to write user feedback when prescribing medications (for example, Reason for patient refusing from medication), reminders or alerts by the system when need to ordering medicines [18], Diagnosis-based selection and diagnostics [13], ability to view a list of suitable drugs with the diagnosis [10, 20], Find and choose the name of drug by the user [15], completion of prescription fields (drug name, dose, route, frequency, prescription instructions, duration of treatment, repetitions) [13, 15], select a set of drug orders from the personal list of drugs previously prescribed by a physician, displaying the profile of the drug [15], Using all available data sources, displaying an overview of current drug treatment and medical services Prescription medications shown in the previous six months, Distribution or non-distribution of prescribed medications, medications provided by other doctors, stoppage of treatment, treatment overlap days By clicking on any drug on your physician's list, you may get information on the cost of medicine, emergency visits and hospitalization, observation details of prescription records, and the average cost of prescriptions in the previous six months. Highlighting of medications regarded as potentially problematic for prescription (based on color code intensity), At least one therapeutic symptom from a list of all authorized or recorded symptoms for each prescription medicine, as well as the possibility to enter unlisted or unlabeled symbols via free text input [22], Simplifying the selection and categorization of drug lists into drug groups [17, 20], providing a selection of available and appropriate dosages and forms for a certaindrug [10, 20], ability to view a list of suitable drugs with the diagnosis [10, 20], custom menus of pharmaceutical options and laboratory tests [10, 17], display of drug options should not be influenced by advertising considerations, access to the meaning of any particular symbol or font must be during the prescribing process [10], integration of cost of drug alternatives [17], determining the real cost to the patient for each drug option based on patient prescription insurance coverage, access to evidence of drug effectiveness and potential harm [10], pharmacological vocabulary according to clinical pharmaceutical form RxNorm [20], immediate access of prescribers due to the choice of drug that the system indicates as recommended or preferred for the current patient [10], Import of side effects of medicines [20]
3	Features, requirements, and recommendations	Security, Privacy and administration [10, 14–16, 18]	Support systems from compliance with state and national privacy regulations [10, 16], Authentication systems for each user, Easy and secure support of clinical data [16, 18], Possibility of correcting or marking the information in the patient file [16], Secure transfer of sensitive data [18], providing reliable mechanisms for regular timely updates from the vendor, require to unique prescriber identifiers [16], control access privileges for individual users or groups of users [10, 16], record and track provision of data to third parties and receiving informed consent for participation in education, research activities, patient support programs and [16], access and evaluation of data by physicians and researchers [23], 24-h user access to IT support in case of failure, high level of security of anti-theft systems [18], Systems should support compliance with the latest standards of portable law and health insurance accountability for privacy and security, each user must be individually identified in the system and have role-based access privileges [10]
4	Features, requirements, and recommendations	Transparency and accountability [10, 14, 16]	Access to information about the techniques used to produce decision support tools and other aspects that may affect the prescriber's decision-making. Access by the user to information regarding the knowledge source(s) used for decision support and other characteristics that may impact the prescriber's decision-making. User access to information about the financial sponsorship used for decision-making. show pharmaceutical company advertisements Commercial factors should not influence how product selections are shown throughout the medicine choosing process [16] The disclosure of Conflict of Interest, electronic prescription system vendors' access to resources and methods used to develop clinical decision support rules, including trigger stimuli and other messages [10]
5	Features	Interoperability and communication [18]	The system interfaces with other software applications for managing patient data [16, 24], supports the use of nationally agreed clinical terminologies and coding systems when they become available, transmits data between service providers using a nationally agreed messaging (content) standard, encrypts all messages transmitted between service providers using a nationally agreed standard, imports and exports a variety of file formats associated with a patient record [16], and electrifies all messages transmitted between service providers. creating an Adverse Drug Reactions Advisory Committee report for a patient and enabling electronic delivery of the report, enabling the prescriber to create and customize templates for letters, referrals, and other documents that can be completed with data from the patient record, to ease medication reconciliation, the system enables the doctor to analyze and reconcile a list of suggested drugs from another source with the patient's existing prescription list [16]
6	Features, requirements, recommendations, and capabilities	Monitoring, report, reminder, and renewals, feedback at the prescriber level [10, 13, 14, 16]	Flaging possible under- or overuse of medications by a patient, reminding to the prescriber when a prescription is due or overdue for a patient, during the consultation, reminding to the prescriber of the need for routine or recommended tests associated with certain medications and conditions, reminding to the prescriber when new test results are available for a patient, tracking clinical orders and reminding to the prescriber when a selected test or investigation was ordered in the recent past, reminding to the prescriber when results are abnormal or require action,reminding to the prescriber when patients are due or overdue for testing, including screening [16], accelerate the renewal process of drugs with the ability to send renewal applications electronically and automate the renewal license process [24], displaying cumulative reports of laboratory test results to facilitate trend analysis, and highlights out of range values, providing a facility to monitor results and clinical outcomes related to specific medications, generating predefined reports to enable clinicians to monitor clinical care and audit individual or practice performance, generating reports based on user defined 'query sets' to monitor clinical care and audit individual or practice performance, generating reports on data for individual prescribers or for all the prescribers in the practice, racking a prescriber's history of alerts that were dismissed, providing reports for producing population-based recalls and reminders, for example in population health programs such as immunisation and epidemiological surveillance, flaging a patient for recall for any reason specified by the prescriber, flaging all medications that were changed at the last consultation, alerting to the prescriber when the patient meets the criteria for a Medication Management Review service [16], Announcement of the time period for version extension [26], Notify me of prescriptions generated as a result of my failure to transmit a prescription to the pharmacist [16], Inform the prescriber if the patient has not got the prescription on time or has received it twice [26], Notification from a pharmacy when a prescription is delivered to a patient, Access to test results [16], Outcome commands (for example, for monitoring experiments) [13], facilitate the renewal of previously prescribed drugs by providing a list of current drugs [15], The feedback and Immediate recommendations to resolve potential issues before order activation [23], review of indexes of self-prescribing patterns by prescribers, profile history of ignored alerts by prescribers [10]
7	Features, requirements, recommendations, and capabilities	Infrastructure: Computer equipment, Awareness of physicians and System support, Patient education and information, Usability [10, 13, 14, 16–18, 20, 21, 23]	The implement computer systems with enhanced capabilities, [23] emphasize training for new physicians and pharmacists [17, 23], providing comprehensive education to all users and patients [10, 14, 18, 24], patient instructions for using the drug at the appropriate education level [10, 20], software technical support [23], eDialog between the prescriber and the pharmacy [17], computer programs user-friendly [23], Quick and easy use of the system [18], facilitate electronic transfer of prescriptions to pharmacies by sending messages and using cooperative standards, print prescription, providing clinical knowledge to the prescriber, improve work processes [24], Transfer discontinuation orders to pharmacies for distribution, Order to stop and change the drug or dose of the drug along with registering the reason for your decision using the drop-down list, the need for pharmacists to know the symptoms of treatment to improve the distribution safety and the need for patient consultation [15], access to information about prescribed medications for patients based on nationally agreed requirements or recommendations, including displaying and printing a complete list of the patient's current medications, providing 'key counseling points' associated with medications and other management options, access to information about medical conditions for patients, and clinical tools for the prescriber to use with a patient, include or exclude the clinical indication as part of the clinical indication [10, 16]
8	Standards	Standards [20, 22, 24]	Formulary and Benefit Standard [20, 22, 24]
			Medication History Standard [22, 24]
			Prescription Fill Status Notification [22, 24]
			RxNorm Standard [20, 22, 24]
			Structured and Codified Sig [20, 22, 24]
			Prior authorization standard [22, 24]
9	Recommendations	History of medications/current medications [10, 14]	Prior to selecting a medicine, access to the patient's medical history is required [16, 20], Extraction of patient data for decision support purposes from external sources such as pharmacies, hospitals, laboratories, and electronic health record (EHR) systems, and over-the-counter and alternative medications prescribed by prescribers [16], Patient flushitis observation (flow chart) in conjunction with a medication history and test results [23], Prescribers may get information on prior individual prescriptions, including dosages, prescription dates, and distribution dates [16], Automatic update of the patient's current medication list [23], Possibility of monitoring patients' regular drug usage and medical treatment outcomes [25]
10	Recommendations and requirements	Data transfer and storage [10, 14]	Send prescriptions to the patient elective pharmacy [10, 23], The transfer of clinical data among systems should comply with the latest versions of Level 7 health standards and/or the National Council for Prescription Drug Programs, Systems should use global provider IDs when available, systems should use global patient identifiers if available, support from the integrity of stored or sent data [10], prescription output by JCAHO requirements for drug naming, abbreviations, etc. [20]
11	Recommendations and capabilities	Alerts and other messages to prescribers, and filtering of user-selectable alerts for possible prescription problems and Decision support [13–16, 18]	Alerts for drug allergies or intolerance and drug interactions [10, 16–20, 23], drug-Test Interference alerts, precautionary alerts for inadvertent errors [20], drug allergy alerts in terms of the food allergies [17, 20], automatic alerts system for contraindications [16, 19, 24], immediate access to the explanation of the rationale of each message [10], distinguish alerts and messages based on patient safety and health concerns from other messages by prescribers, prioritizing safety alerts based on clinical significance [10, 16, 18, 24], suppression of low-priority safety alerts by the prescriber to reduce false positive alerts [10, 23], providing the possibility customization of alerts by the system [16, 18], automated alerts for duplicates therapies and prescriptions [16, 19, 20, 23], system alert for overdose [19, 23], system alert for dose adjustment based on age, sex, weight and kidney and liver function of patients [10, 16, 19, 20], system alert to physician when laboratory test results need to be followed up [10], timely reminders of upcoming monitoring events schedule [20], alerts and messages should indicate the date of the last update of Basic Decision Support Rules [10], The assessment of possible prescribing problems related to drug-contraindications for a particular patient selected, the assessment of possible prescribing problems related to age, medication and age of drug-allergy for selected patient, assessment of possible prescribing problems related to drug interactions for a selected specific patient [13, 15], the assessment of possible prescribing problems related to the repeated therapy for selected patient, the assessment of possible prescribing problems related to the cumulative toxicity for specially selected patient [15], safety alerts base on laboratory medicine (kidney, liver function), safety alerts based on body size, age (child, adult, …), safety alerts based on drug formulation [13], classification of levels of severity of alerts into three levels of complete prohibition, should be avoided if possible, and used with caution, possibility to filter alerts by physicians selectively according to the intensity of alerts, stop alerts that physicians deem clinically irrelevant, display alerts when opening patient records, viewing drug profiles and creating prescriptions [15], alert for fulfillment of patients [13], documenting the reason for ignoring the warning from the drop-down menu by the physician (for example, more benefit than risk) [15], alert for a woman who is pregnant or breastfeeding, alert for patients whose status is recorded as 'elite athlete', the system alert when a prescription for a new or alternative medication is indicated, based on data in the patient record and national best practice guidelines, the system distinguishes alerts and messages based on patient safety, health outcomes, and cost effectiveness concerns from those based on advertisements and other commercial considerations, direct access to the patient data that triggered an alert, suppression of alerts by users, decision support information ​when it was last updated, the system alerts the prescriber when two patients in the system have the same name, during the creation of a new record or when opening an existing record, a preview and reminds the user to confirm that the identity of the patient and the details of prescription, referral or other order prior to electronic transmission, reminder to the user for fulfil regulatory requirements for Pharmaceutical Benefits Scheme† and/or other restricted medications [16], enter additional information about patient medications by the user, The possibility of prescribing unlicensed drugs [18]

**Fig. 2:**
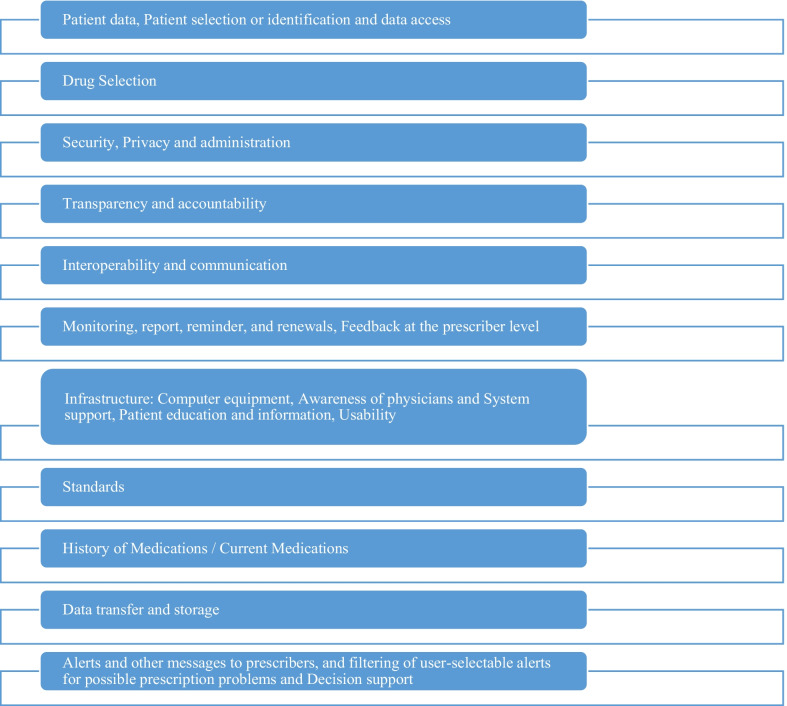
Recommendations for improving the electronic prescription system

Recommendations for improving the electronic prescription system see in Fig. 2 (Fig. 2).

## Discussion

The purpose of this research was to use the scoping review approach to ascertain the needs for an electronic prescription system. The findings of this research indicate that the following needs should be addressed while developing an electronic prescription system: Patient data, Patient selection or identification and data access, Drug Selection, Security, Privacy and administration, Transparency and accountability, Interoperability and communication, Monitoring, report, reminder, and renewals, Feedback at the prescriber level, Infrastructure: Computer equipment, Awareness of physicians and System support, Patient education and information, Usability, Standards, History of Medications / Current Medications, Data transfer and storage, Alerts and other messages to prescribers, and filtering of user-selectable alerts for possible prescription problems and Decision support. One of the requirements in this study is patient identification, which is usually the first step of electronic prescribing [13]. Prescribers often make mistakes when choosing from menus, and as a result, inadvertently choose the wrong patient. Errors are reduced when there is a detection and correction system [25]. Since the reduction of errors in the electronic prescription system is one of the main advantages of this system, so the correct identification of the patient is one of the main requirements.

One of the requirements of electronic prescription system identified in this study is safety alerts and filtering of user-selectable alerts for possible prescribing problems. Safety alarms in the system dramatically reduce allergy errors and drug selection [26]. Adequacy of system alerts should be considered as one of the main requirements. Repeated and excessive error messages are likely to reduce users' sensitivity to them. Moreover, when there is a reasonable reason for the warning displayed, prescribers will no doubt accept a higher percentage of drug interaction alerts.

Another requirement that we achieved in the present study was computer-assisted dose calculations, increasing prescribing accuracy [27, 28]. However, appropriate calculations require electronic prescription systems to access medical records data such as age, weight, BMI, and laboratory results that reflect renal and hepatic function [29].

Data transfer and storage were identified as one of the recommendations to improve the electronic prescribing systems. Transmitting data electronically from prescribing systems to pharmacies led to eliminating human transcription errors, and improving safety and efficiency. However, errors or physician work might increase if transmissions are unreliable or if prescribing data is entered manually at the pharmacy [13]. Proper transfer of data to the pharmacy or in other words entering the electronic prescription by the physician (instead of the prescription being written by the physician and the pharmacy entering the pharmaceutical items in the system) is necessary, especially in the countries that have recently worked with this system.

Providing infrastructures, such as computer equipment, system support, patient education, information, and user education were identified as features of the electronic prescription system. Well-designed training materials can reduce outpatient errors. The systems which facilitate physician-nurse-pharmacist collaboration in patient education can increase compliance [30]. Unfortunately, in developing countries, because of users' resistance to change, their educability is affected. On the other hand, the low speed of the national Internet in such communities is one of the underlying causes for the failure of projects. Therefore, much attention to the main infrastructure of this system is a key factor.

The research cited prescriber input as a guideline for enhancing electronic prescription systems. Prescription systems that have access to pharmacy data may alert doctors when patients fail to complete prescriptions on time, allowing physicians to probe patient non-compliance. However, physicians presently lack this capability [31, 32]. Numerous computerized tools, like as reminders, feedback, and treatment suggestions, as well as patient care based on established protocols, may help physicians improve their prescription [33].

Data security and confidentiality were identified as the key requirements in the implementation of electronic prescription systems. Security and privacy are two key challenges that electronic health systems face [34]. Security of medical data can be controlled easily by healthcare organizations; however, if medical data is to be transmitted to some other healthcare institution then some third party may compromise with the security and privacy of medical data [35]. There is no doubt that patient privacy is necessary, but with the emergence of various applications that help users to better implement electronic prescribing should be accompanied by caution and compliance with legal issues.

Another requirement identified in the present study was the drug selection and the history of current drugs/drugs in the electronic prescription system. Drug lists included in e-prescriptions should be precise and clear, and the system should include patient follow-up about medications that were previously prescribed [36]. Electronic prescription is suggested to improve proper medication adherence and access to medication history [24]. The physician's knowledge of the patient's medical history, especially in the case of chronic or special patients, is particularly important.

Another essential requirement of the electronic prescription system that we achieved in this study was decision support. Clinical decision support in electronic prescribing systems that provide physicians or patients with clinical knowledge and are presented at appropriate times can improve the safety, quality, efficiency, and cost-effectiveness of care. However, these potential benefits have not been fully realized [20]. Of course, decision support seems to act as a double-edged sword. Because on the one hand, it guides and helps the doctor in making decisions, and on the other hand, it confronts the doctor with limitations. In other words, the physician's decision is influenced by the support system and he cannot diagnose and treat independently. While, the system may not be able to cover a wide range of medical science.

Transparency and accountability were identified as other important requirements for the implementation of an electronic prescription system. Wang et al. panel’s recommendations included several related to transparency and accountability in the electronic prescription system, which prevent third parties from introducing prescribing biases that would not benefit patients, because vendors can substantially influence prescribing decisions [10]. Transparency in system alerts and messages is an essential part of electronic prescription and should be such that there is no conflict of interest.

The standards for the implementation of an electronic prescription system were identified. Formulary and Benefit (F&B) standard provides data for drug insurance benefits plans as opposed to data about individual patients which is necessary to enable the display of coverage information for each medication in the pick-lists that prescribers use to make initial medication choices. SCRIPT standard provides prescribers with information about patients’ current and past medications by listing the pharmacy claims that the patient's health plan has paid for. SureScripts now enforce this standard to list drug purchases beyond what is paid for by insurance. The prescription fill status notification standard is rarely used among electronic prescription systems. This transaction, initiated by the pharmacy, is designed to inform the prescriber of pharmacy events, including distribution, partial distribution, or non-distribution for the original prescription and refill. Prior authorization (PA) standard in electronic prescription systems refers to the process of requesting approval for a prescription’s coverage from the health plan or PBM [22]. RxNorm standard is a drug nomenclature that was created by the National Library of Medicine to standardize the representation of clinical drugs, distinguishing drugs based on their therapeutic or diagnostic intent [8, 22]. The Structured and Codified Sig standard is intended to provide an interpretable representation for the patient instructions portion of a prescription, thereby enabling more automated safety checking, improved communication between prescribers and pharmacists, and better efficiency of prescribing, renewal, and dispensing activities [22]. Wang et al. findings indicate that federal standards for electronic prescribing could best advance patient safety, health outcomes, and health care efficiency by including a minimal set of functional capabilities along with the more technical standards for system interoperability [10].

## Limitations

This study had its limitations. We could not access the full text of some of the studies. Although we contacted the authors by email and telephone, we did not have access to the full text of their articles. Another limitation of this study was the lack of university access to the Embase database, so this database was not searched in terms of the lack of subscription at the university.

## Conclusion

This paper discusses the prerequisites for implementing electronic prescription systems correctly, accurately, and completely. The findings of this study indicated that existing electronic prescription systems possess a variety of functional and technical capabilities that, when properly identified and utilized, can result in significant benefits for all system stakeholders, including service providers, drug distributors, patients, and insurance organizations. It is advised that each of these needs be carefully considered when designing electronic prescription systems to ensure their quality and safety. Additionally, legislators, decision-makers, and insurance companies may utilize the set of needs established in this research to build assessment criteria for the electronic prescription system.

## Data Availability

All data generated or analysed during this study are included in this published article.
